# Genomic resources for *Myzus persicae*: EST sequencing, SNP identification, and microarray design

**DOI:** 10.1186/1471-2164-8-423

**Published:** 2007-11-16

**Authors:** John S Ramsey, Alex CC Wilson, Martin de Vos, Qi Sun, Cecilia Tamborindeguy, Agnese Winfield, Gaynor Malloch, Dawn M Smith, Brian Fenton, Stewart M Gray, Georg Jander

**Affiliations:** 1 Boyce Thompson Institute for Plant Research, Tower Road, Ithaca, NY 14853, USA; 2 Department of Biology, University of Miami, Coral Gables, Florida 33146, USA; 3 Department of Ecology and Evolutionary Biology, University of Arizona, Tucson, Arizona 85721, USA; 4 Cornell Theory Center, Cornell University, Ithaca, NY 14853, USA; 5 USDA/ARS, Plant Protection Research Unit, Ithaca, NY 14853, USA; 6 Scottish Crop Research Institute, Invergowrie, Dundee, UK

## Abstract

**Background:**

The green peach aphid, *Myzus persicae *(Sulzer), is a world-wide insect pest capable of infesting more than 40 plant families, including many crop species. However, despite the significant damage inflicted by *M. persicae *in agricultural systems through direct feeding damage and by its ability to transmit plant viruses, limited genomic information is available for this species.

**Results:**

Sequencing of 16 *M. persicae *cDNA libraries generated 26,669 expressed sequence tags (ESTs). Aphids for library construction were raised on *Arabidopsis thaliana*, *Nicotiana benthamiana*, *Brassica oleracea, B. napus*, and *Physalis floridana *(with and without *Potato leafroll virus *infection). The *M. persicae *cDNA libraries include ones made from sexual and asexual whole aphids, guts, heads, and salivary glands. *In silico *comparison of cDNA libraries identified aphid genes with tissue-specific expression patterns, and gene expression that is induced by feeding on *Nicotiana benthamiana*. Furthermore, 2423 genes that are novel to science and potentially aphid-specific were identified. Comparison of cDNA data from three aphid lineages identified single nucleotide polymorphisms that can be used as genetic markers and, in some cases, may represent functional differences in the protein products. In particular, non-conservative amino acid substitutions in a highly expressed gut protease may be of adaptive significance for *M. persicae *feeding on different host plants. The Agilent eArray platform was used to design an *M. persicae *oligonucleotide microarray representing over 10,000 unique genes.

**Conclusion:**

New genomic resources have been developed for *M. persicae*, an agriculturally important insect pest. These include previously unknown sequence data, a collection of expressed genes, molecular markers, and a DNA microarray that can be used to study aphid gene expression. These resources will help elucidate the adaptations that allow *M. persicae *to develop compatible interactions with its host plants, complementing ongoing work illuminating plant molecular responses to phloem-feeding insects.

## Background

Insects in the order Hemiptera, which includes all insects that feed exclusively or predominantly on phloem sap, currently represent the most significant challenge for agricultural pest management programs. Although transgenic plants producing *Bacillus thuringiensis *(Bt) toxin have achieved resistance to many devastating lepidopteran pests, these crops remain susceptible to infestation by aphids and other hemipterans. Reduction in insecticide application, concomitant with the widespread cultivation of Bt crops, has resulted in hemipteran pests being the primary insect threat in major agricultural systems [1]. Aphid feeding causes an alteration of plant source-sink relationships [2], the induction of premature leaf senescence [3], secondary pathogen infection through fungal growth on aphid honeydew, and the transmission of plant viruses [4]. Among these, virus transmission by aphids represents the greatest threat for agricultural crops. *Myzus persicae *(green peach aphid), which is capable of transmitting more than 100 plant viruses, is the world's most versatile aphid viral vector [5,6]. In particular, *M. persicae *is a very efficient vector of *Potato leafroll virus *(PLRV), which can lead to yield reductions of 40–70% in infected fields [7]. *M. persicae *lineages can vary considerably in their PLRV transmission efficiency [8], suggesting that there are lineage-specific genetic factors that influence this trait.

*M. persicae *has been found on hundreds of mostly dicotyledonous plant species [6]. Given this broad host range, it is not surprising that differences in host plant utilization among *M. persicae *lineages are quite common, and efforts have been made to identify molecular variation that correlates with host range and reproductive life cycle [9,10]. The best-studied example of such variation is represented by the typically red-colored lineages that are able to thrive on tobacco [11]. These first appeared on tobacco in Japan more than 60 years ago and have spread to all tobacco-growing regions of the world. In the United States, *M. persicae *has been found on tobacco since at least 1947 [12]. Red strains of *M. persicae *were first reported in the United States in 1985, and by 1987 had become the dominant color morph on tobacco [13,14]. The tobacco-adapted lineage of *M. persicae *has been granted subspecific status, *Myzus persicae nicotianae *[15].

In the laboratory, several plant species are convenient hosts for rearing and studying *M. persicae*. *Brassica oleracea *(cabbage) is commonly employed as a host plant for maintaining aphid cultures. *Arabidopsis thaliana *(Arabidopsis), a well-developed model genetic organism with a fully-sequenced genome, is readily consumed by *M. persicae*, and *A. thaliana *microarray studies have identified genes that are induced or repressed in response to *M. persicae *feeding [16-20]. *Nicotiana benthamiana*, a wild relative of tobacco [21] also serves as a host plant for some lineages of *M. persicae*. Virally induced gene silencing (VIGS) is particularly effective in *N. benthamiana*, permitting rapid screening of individual genes to study their importance in defense against *M. persicae *and other herbivores [22]. *P. floridana *(downy ground-cherry) serves as a model solanaceous plant for studying the transmission of PLRV by *M. persicae *[23].

So far, only limited sequence information and genetic markers are available for the estimated 313 Mb nuclear genome of *M. persicae *[24-27]. However, recent advances in DNA sequencing make it possible to rapidly acquire information about the coding regions of any genome by building complementary DNA (cDNA) libraries and sequencing expressed sequence tags (ESTs). Here we describe the creation of such an EST database from 16 sequenced *M. persicae *cDNA libraries and the use of these data to make *in silico *predictions of differentially expressed genes, identify single nucleotide polymorphisms (SNPs) between lineages, and develop probes for an oligonucleotide microarray to study aphid gene expression.

## Results

### Phenotypic Characterization of *M. persicae *Lineages

Phenotypic characterization and microsatellite genotyping of lineage 2001–12, which was collected in Scotland, has been described previously as clone Type B [28]. Other *M. persicae *lineages were collected at five sites in the United States, and were characterized to determine their reproductive life cycle (Additional file 1). Of the 46 tested aphid lines, eight were holocyclic, five intermediate, 15 androcyclic, and 17 anholocyclic. One lineage remained unclassified because multiple replicates failed to grow. Seven holocyclic *M. persicae *lineages and one anholocyclic tobacco-adapted lineage were genotyped with seven microsatellite markers, and were determined to be genetically distinct (Table 1).

**Table 1:** Life cycle and microsatellite genotype of *M. persicae *lineages

						*Microsatellite fragment sizes (base pairs)						
						
**Lineaggege**	**Location**	**Date**	**Host**	**Color**	**Lifecycle**	**M86**	**S17b**	**myz25**	**myz3**	**M40**	**myz2**	**myz9**
F001	Freeville, New York	8/20/03	squash	green	Holocyclic	117 117	161 161	116 142	121 121	123 135	192 206	195 195
F009	Freeville, New York	8/20/03	Potato	green	Holocyclic	111 129	165 165	116 118	Null	127 127	188 190	209 223
F012	Freeville, New York	8/20/03	Potato	green	Holocyclic	129 131	165 165	118 118	121 121	123 123	192 202	193 203
G002	Geneva, New York	8/19/03	Pepper	green	Holocyclic	91 91	161 161	116 118	123 123	123 127	178 178	203 223
G003	Geneva, New York	8/19/03	Pepper	green	Holocyclic	Null	161 165	116 142	123 123	Null	192 202	209 209
G006	Geneva, New York	8/19/03	Pepper	green	Holocyclic	117 117	Null	116 120	121 121	123 133	190 202	221 221
G010	Geneva, New York	8/19/03	Pepper	green	Holocyclic	117 117	165 167	120 142	121 123	123 123	202 206	195 195
USDA	Ithaca, New York	2003	Tobacco	red	Anholocylic	93 101	165 165	118 118	121 121	115 123	192 202	215 219
2001–12	Dundee, Scottland	7/4/01	Potato	red	ND	ND	ND	ND	ND	ND	ND	ND

* Two different fragment sizes indicate heterozygosis at this marker; Null = no amplification; ND = not determined.

Seven holocyclic *M. persicae *lineages (Table 1) were tested for their ability to transmit PLRV acquired from detached virus-infected leaves of *P. floridana *to virus-free *P. floridana *plants. In three independent trials, aphids of clone G006 transmitted PLRV efficiently, whereas aphids from lineage F001 failed to transmit the virus consistently (Table 2). In these experiments, the F001 and G006 clones exhibited similar growth rates and fecundity, suggesting that the observed differences in transmission are attributable to differences in the clones' capacity to acquire and/or transmit the virus, rather than to differences in the amount of time spent feeding on the infected or uninfected leaves. In control experiments, aphids from both lineages that were transferred from uninfected detached leaves to uninfected plants failed to transmit PLRV. The USDA aphid lineage, which was used as a positive control, transmitted PLRV with 100% efficiency in these experiments (data not shown).

**Table 2:** PLRV transmission by holocyclic *M. persicae *lineages.

	**% Transmission**		
	
**Clone**	**Trial #1**^a^	**Trial #2**^a^	**Trial #3**^b^
G002	40	60	-
G003	80	-	-
G006^c^	100	100	100
G010	100	-	-
F001^c^	40	0	10
F009	80	-	-
F012	40	40	17

^a^Five plants per experiment; ^b^Ten plants per experiment, except six plants for F012; ^c^Lineages used for cDNA library construction

### cDNA Library Construction and Sequencing

**Figure 1:**
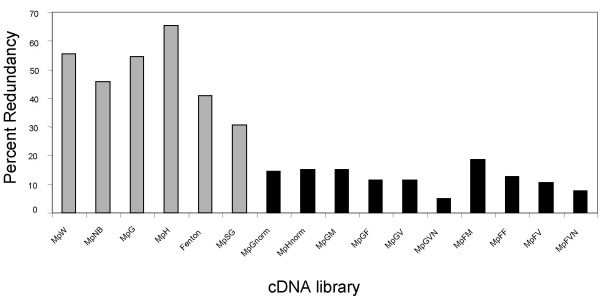
Percent redundancy of *M. persicae *cDNA libraries. For each library, percent redundancy = 100 × [1-(#unigenes)/(#ESTs)]. Gray bars represent non-normalized libraries; black bars represent normalized libraries.

As summarized in Table 3, 16 cDNA libraries representing a diversity of tissues and developmental stages were constructed from four aphid lineages (USDA, 2001–12, F001, and G006). Aphids were reared on host plants from the Solanaceae and Brassicacae families, as well as on plants with and without PLRV infection. Since sequencing non-normalized libraries showed a high level of redundancy, normalized cDNA libraries were created to improve the rate of new gene discovery (Fig. 1). Although normalization increased the gene discovery rate, it also precluded making inferences about differential gene expression by comparing EST frequencies between these libraries. Altogether, sequencing of the cDNA libraries produced a total of 26,759 high quality sequencing reads, which have been submitted to GenBank (accession numbers: DW010205–DW015017, EC387039–EC390992, EE570018–EE572264, EE260858–EE265165, ES444641–ES444705, ES217505–ES226848, and ES449829–ES451794).

**Table 3:** Description of source tissue for each of 16 *M. persicae *cDNA libraries.

**Library**	**Clone**	**Tissue Type**	**Host Plant**	**Normalized?**	**ESTs**	*Unigenes
MpW	USDA	Whole body, asexual females	*Arabidopsis thaliana*	No	4798	2136
MpNB	USDA	Whole body, asexual females	*Nicotiana benthamiana*	No	1020	552
MpG	USDA	Digestive Tract	*Arabidopsis thaliana*	No	750	340
MpH	USDA	Head	*Arabidopsis thaliana*	No	746	259
MpSG	USDA	Salivary Glands	*Brassica oleracea*	Yes	3233	2242
MpGnorm	USDA	Digestive Tract	*A. thaliana/N. benthamiana*	Yes	1807	1542
MpHnorm	USDA	Head	*A. thaliana/N. benthamiana*	Yes	2063	1753
Fenton	2001–12	Whole body, asexual females	*Brassica napus*	No	2019	1196
MpGM	G006	Whole body, males	*Brassica oleracea*	Yes	1437	1219
MpGF	G006	Whole body, sexual females	*Brassica oleracea*	Yes	1388	1227
MpGV	G006	Whole body, PLRV infected asexual females	*Physalis floridana*	Yes	1299	1150
MpGVN	G006	Whole body, PLRV free asexual females	*Physalis floridana*	Yes	866	822
MpFM	F001	Whole body, males	*Brassica oleracea*	Yes	1359	1106
MpFF	F001	Whole body, sexual females	*Brassica oleracea*	Yes	1294	1129
MpFV	F001	Whole body, PLRV infected asexual females	*Physalis floridana*	Yes	1328	1189
MpFVN	F001	Whole body, PLRV free asexual females	*Physalis floridana*	Yes	1262	1164
				
				**All Libraries**	**26,669**	**10,341**

*For each library, the number of contigs and singletons is combined to indicate how many unigenes are represented in the library. As many contigs are generated by aligning ESTs from multiple libraries, the total number of unigenes from all libraries is less than the sum of the number of unigenes from each individual library.

### Sequence Assembly and Annotation

**Figure 2:**
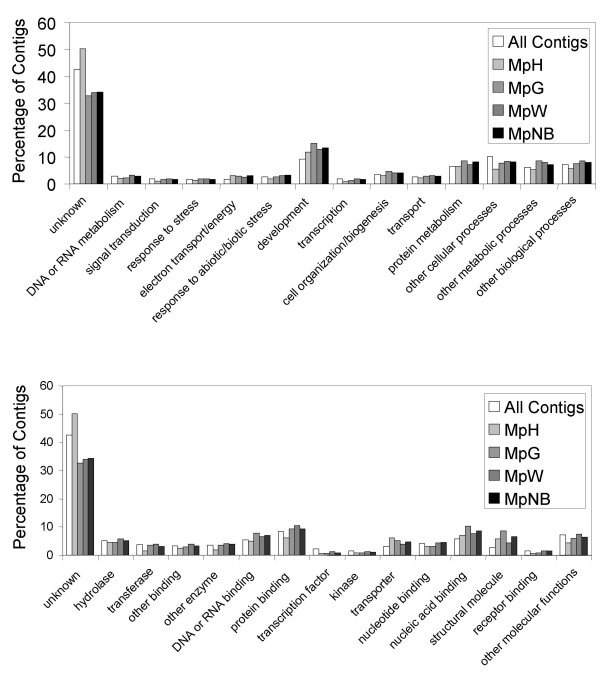
(A) Distribution of Gene Ontology biological process annotations, compared between non-normalized libraries. (B) Distribution of Gene Ontology molecular function annotations, compared between non-normalized libraries.

We identified 3965 contigs and 6376 singletons in the 26,759 high-quality sequences. BlastX (E-value cutoff = 1E-5) was run on all 10,341 unigenes against a database containing all NCBI RefSeq proteins plus the 105 *M. persicae *proteins available at the time in GenBank (January 25, 2007). A one-line annotation was generated for each contig in the following way: (i) if the best Blast hit was for a known *M. persicae *protein, that annotation was used; otherwise (ii) if there was a hit for a *Drosophila melanogaster *protein, that annotation was considered to be most reliable; and (iii) if there were no hits to *D. melanogaster *or *M. persicae *proteins, we used the annotation of the best Blast hit. In addition, the top ten Blast hits are listed for each contig in Additional file 2. Information on the ESTs from which each contig is built, including GenBank accession number and source library, is provided (Additional file 3). GOslim annotations [29] were tabulated for each contig, and a summary of the molecular function and biological process annotation is provided for the total EST collection and separately for the ESTs from the non-normalized libraries (Fig. 2).

The top twenty contigs in terms of representation in the EST collection can be found in Table 4, ranked according to the number of ESTs they contain. Seven of the most highly expressed genes in *M. persicae *have no significant similarity to any proteins in the RefSeq database [30]. We compared our sequences to the mitochondrial genome of the aphid *Schizaphis graminum *(greenbug) and identified 880 ESTs likely to represent mitochondrial genes. Of these, 491 constitute four contigs that are among the most highly expressed genes in our database (Table 4).

**Table 4:** RefSeq annotation for the twenty *M. persicae *contigs composed of the largest number of ESTs.

**Rank**	**Contig ID**	**Number of ESTs**	**Gene Description**	**Predicted Mitochondrial Or Nuclear Location**
1	1	268	12S small subunit ribosomal RNA gene	Mitochondrial
2	1194	155	unknown protein	Nuclear
3	2319	148	cytochrome c oxidase subunit III	Mitochondrial
4	3100	100	unknown protein	Nuclear
5	3675	67	ATP synthase F0 subunit 6	Mitochondrial
6	3801	60	ribosomal protein, large subunit	Nuclear
7	3543	59	cytochrome c oxidase subunit I	Mitochondrial
8	8	59	tentative cuticle protein	Nuclear
9	130	51	unknown protein	Nuclear
10	375	47	unknown protein	Nuclear
11	1079	43	unknown protein	Nuclear
12	1313	42	ribosomal protein S11	Nuclear
13	844	40	unknown protein	Nuclear
14	1429	39	ribosomal protein L10	Nuclear
15	3408	39	unknown protein	Nuclear
16	1768	37	ribosomal protein L11	Nuclear
17	1881	37	ribosomal protein S24	Nuclear
18	731	37	ribosomal protein S8	Nuclear
19	495	37	muscle LIM protein	Nuclear
20	254	36	cathepsin B	Nuclear

We compared the 10,341 EST contig sequences of *M. persicae *with 17,069 contigs of *Acyrthosiphon pisum*. Using BLASTn (E-value cutoff = 1E-10), there were 5513 *A. pisum *contigs with BLAST hits in *M. persicae *contigs, and 5598 *M. persicae *contigs with BLAST hits in *A. pisum *contigs. The low overlap between the contig sequences may be due to the fact that neither EST database represents the whole transcriptome. However, it is likely that many of the contigs in one aphid which do not have homologues in the database of the other aphid species may represent genes responsible for the adaptation to specific host plants, or for other differences in physiology which have evolved since the species' divergence. There is significant similarity between predicted coding regions in a subset of *M. persicae *and *A. pisum *genes. From more than 5000 shared contig sequences, 1585 have greater than 95% identity in the coding region (coding regions are defined as open reading frames of 50 residues or longer that can be aligned to Uniprot sequences with BlastX E-values less than 1E-10). These genes are likely to represent cellular housekeeping genes, but may also include genes which are essential for aspects of plant-insect interactions which are specific to, and common among, aphids.

### Comparision of *M. persicae *ESTs to other Genomic Data

Similarity searches against other aphid ESTs, performed using the TBlastX program (E-value cutoff = 1E-10), identified 4500 unigenes with no similarities to previously described aphid ESTs. Therefore, these unigenes represent newly described aphid cDNA sequences. However, some of these sequences may arise from untranslated regions of genes, which may not be highly conserved between species. These 4500 unigenes were subsequently compared with the *nr *database (non-redundant NCBI protein and nucleotide database) using TBlastX and BlastX programs. A total of 2423 unigenes had no hits at E-value <1E-10. Some subset of these may represent *M. persicae*-specific genes. The other 2077 unigenes represent "new-to-aphids" features: genes identified in non-aphid species, which are not represented among the over 80,000 ESTs from five other aphid species that were previously submitted to GenBank.

Blasting the 959 *M. persicae *ESTs previously described by Figueroa *et al*. [27] against our database revealed that 806 of these ESTs match up to 520 of our unigenes at E-values<1E-100. Based on this stringent cut-off value, 153 of these previously described *M. persicae *ESTs were not found in our data set – however, 109 of these ESTs have matches at E-values<1E-20, indicating that they may represent closely related gene family members.

BlastN (E-value cutoff = 1E-20) of all ESTs against the three *Buchnera aphidicola *genomes available in GenBank, as well as BlastX (percentage identity cutoff 80%) against all *Buchnera aphidicola *protein sequences in GenPept identified 90 sequences that are almost certainly from the bacterial endosymbiont of *M. persicae, Buchnera aphidicola*. The top Blast hit for five of our unigenes (all singletons) are for *B. aphidicola *proteins, indicating that our filtering failed to remove a small number of contaminating bacterial sequences. No *Escherichia coli *or *Saccharomyces cerevisiae *proteins appeared as a top-ten Blast hit for any of our unigenes giving us confidence that significant contamination from these sources is not a concern.

### *In Silico *Prediction of Differentially Expressed Genes

We used the previously described R statistic [31] to identify the contigs showing the greatest differences in EST abundance among four of the non-normalized libraries (MpH, MpG, MpNB, MpW). A log likelihood ratio statistic was calculated that estimated the extent to which differences in gene expression correspond to the heterogeneity of the libraries. The twenty top hits of differentially expressed genes are presented with a brief description of the protein, the value of the R statistic, and the abundance of the gene in each of the four libraries (Table 5). Among the twenty contigs showing the highest R value, twelve represent genes that are over-expressed in the head library. None of these genes show similarities to published proteins with known function (E-value cutoff = 1E-5), and ten of the twelve genes were found only in head or full body cDNA libraries made from *M. persicae *or other aphids. The six contigs representing genes that were most highly expressed in the gut library include two with no homology to GenBank sequences. Two other contigs show similarity to the lysosomal cysteine protease cathepsin B-N. Contig 3427 shows similarities to a structural protein from densoviruses, which have recently been described as infecting the stomach cells of aphids [32]. Contig 1196, which represents a gene that is more highly expressed in the gut, shows similarity to a glutathione S-transferase (GST). GSTs belong to a large family of proteins implicated in xenobiotic detoxification, and an increase in GST activity has been associated with the adaptation to plant secondary metabolites in *M. persicae *[33]. Two contigs with no homology to known genes have a significant overrepresentation of ESTs from aphids reared on *N. benthamiana *rather than *A. thaliana*. One of these contigs, number 1079, also contains five ESTs from the digestive tract library and none from the head library, suggesting this gene as a candidate for involvement in aphid response to tobacco-specific defenses.

**Table 5:** USDA lineage contigs with library-specific expression patterns in non-normalized libraries.

**Contig ID**	**MpG**^a^	**MpH**^b^	**MpNB**^c^	**MpW**^d^	**Total ESTs**	**R**_tot _^e^	**Homology**	**E-value**
1079	5	0	**28**	9	42	34.4	unknown protein	NA
3260	0	1	**18**	2	21	28	unknown protein	NA
8	0	**42**	1	13	56	68.2	unknown protein	NA
3100	0	**35**	0	5	40	66.9	unknown protein	NA
613	0	**28**	1	1	30	57.5	unknown protein	NA
375	0	**17**	0	1	18	35.3	unknown protein	NA
3414	0	**15**	0	0	15	34.2	unknown protein	NA
2753	0	**18**	0	3	21	33.7	unknown protein	NA
3104	0	**20**	1	6	27	31.8	unknown protein	NA
3194	0	**17**	0	3	20	31.6	unknown protein	NA
3319	0	**13**	0	2	15	24.6	unknown protein	NA
2148	0	**10**	0	0	10	22.8	unknown protein	NA
614	0	**9**	0	0	9	20.5	unknown protein	NA
844	0	**10**	0	3	13	17	unknown protein	NA
1196	**23**	0	0	2	25	46.2	glutathione S transferase S1	1E-53
130	**33**	0	3	15	51	46.1	unknown protein	NA
448	**16**	0	0	1	17	33	unknown protein	NA
254	**20**	0	0	11	31	30	cathepsin B-N	0
3427	**16**	0	1	2	19	29	densovirus structural protein	1E-46
256	**9**	0	0	0	9	20.5	cathepsin B-N	1E-161

^a^MpG = digestive tract (gut) library; ^b^MpH = head library; ^c^MpNB = whole body library, reared on *N*. benthamiana; ^d^MpW = whole body library, reared on *A. thaliana*; ^e^R_tot _= likelihood ratio statistic [34].

### Prediction of Secreted Salivary Proteins

In order to find aphid proteins involved in the successful infestation of host plants, we have identified cDNA sequences expressed in the salivary glands that are predicted to encode for secreted proteins. These proteins may be required for the establishment of prolonged phloem feeding and suppression of plant defenses. Using stringent criteria (see Materials and Methods) we identified 186 contigs representing sequences expressed in salivary glands. Subsequent *in silico *translation and signal peptide prediction resulted in the identification of 45 *M. persicae *proteins that may be secreted from the salivary glands (Additional file 4). These include a total of fifteen proteins that are predicted to possess an anchor sequence (Additional file 4), indicating that these proteins remain in the cell membrane upon secretion and might function as receptors or proteins involved in transport. For instance, the protein encoded by contig 515, is a close homologue of tetraspanin 29FA in *D. melanogaster*, where it functions as a cell surface receptor binding protein involved in signal transduction (Mi et al., 2003 – personal communication to FlyBase, ).

### SNP Identification and Validation

SNPs are effective molecular markers for genetic mapping and can also be used to estimate the level of sequence divergence between lineages. Using the program PolyBayes [34], we identified 12,722 potential SNPs from our EST sequences. Since we were interested in identifying SNPs that represented differences between, rather than within, lineages, we filtered our list of polymorphisms to include only those SNPs representing a nucleotide difference between two lineages, and exhibiting no apparent heterozygosity within lineages. This resulted in ~800 polymorphisms that can serve as potential molecular markers for differentiating aphid lineages (Additional file 5).

Many of the predicted SNPs are represented by only a single EST in one or more lineage, allowing for the possibility that observed sequence differences are artifacts resulting from an error in reverse transcription of the mRNA, PCR amplification of the cDNA, or the sequencing reaction. Therefore, we generated a list of high-confidence polymorphisms in which each of any two lineages was represented by two or more ESTs with the same base at the polymorphic position. This resulted in 167 high-confidence SNPs (Additional file 6), from which we selected a small subset to validate by re-sequencing genomic DNA from *M. persicae *lineages USDA, F001, and G006. Eleven SNPs from seven contigs were selected for validation – contigs contained either one, two, or three predicted polymorphisms (Table 6). No sequence differences between these lineages were detected when sequencing 158 bp of a control gene, EF-1α (accession numbers EF660853–EF660855). Five of the 11 tested SNPs were confirmed by resequencing (Table 6). Four of these confirmed SNPs represented differences between the two green aphid lineages, F001 and G006, and the red tobacco-adapted USDA lineage. Moreover, three of these SNPs were in the open reading frame of contig 254, which is annotated as a lysosomal cysteine protease cathepsin B-N.

**Table 6:** SNP validation by re-sequencing chromosomal DNA

**Contig ID**	**SNP ID**	**Base Position**	**Validated**
129	1849	230	No
254	7292	1023	Yes
254	7293	1045	Yes
254	7294	1065	Yes
1080	543	850	Yes
3202	11100	409	No
3202	11103	554	No
3285	11751	162	No
3285	11752	204	No
3347	12372	256	No
3713	15695	228	Yes

**Figure 3:**
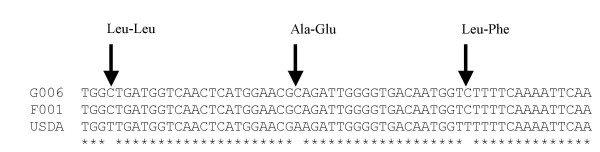
SNPs in contig 254, cathepsin B, a putative gut-specific cysteine protease. Three SNPs were validated by sequencing genomic DNA. Three SNPs in the gene result in one silent change (Leu-Leu), one substitution of a negatively charged amino acid for an aliphatic amino acid (Ala-Glu) and one substitution of an aromatic for an aliphatic amino acid (Leu-Phe) in the green *M. persicae *lineages (G006 and F001) relative to the red USDA lineage.

Contig 254 is one of the most highly expressed genes (Table 4) and differentially expressed in the aphid digestive tract (Table 5). Two of these cathepsin SNPs represent non-synonymous, non-conservative amino acid changes (Fig. 3), indicating a possible functional change in enzyme activity. Structural modeling with the protein fold recognition program Phyre [35] indicates that these residues are in a conserved region in an exposed loop on the surface of the protein. One EST from the second red *M. persicae *lineage (2001–12), shared the three polymorphic nucleotides with the red USDA lineage. Therefore, it may be informative to genotype a wider range of red and green aphids at this locus to determine whether these polymorphisms correlate with differences in host range or life cycle between lineages. Although it is tempting to speculate that this gut-specific protease is undergoing rapid evolution in order to avoid plant protease inhibitors, the small size of our SNP dataset and the high (>50%) occurrence of false positives prevent us from inferring the significance of changes in this protein arising from variation between these lineages.

### Microarray Design

The Agilent eArray platform was used to design a microarray based on our ESTs and an additional 1,121 ESTs from other sources that were available in GenBank (including [27]. Of the total of 10,525 unigenes assembled from these ESTs, we successfully designed 60-mer probes for 10,478 using eArray software. For >95% of the unigenes, three 60-mer probes were designed, corresponding to different regions of the gene. The actual synthesized array consists of one probe group representing all 10,478 unigenes, a second probe group with alternate 60-mers for 4139 of the unigenes, 11 ESTs from *Schizaphis graminum *(greenbug), negative controls corresponding to plant and human specific genes, and positive controls representing insect housekeeping genes. The current slide layout consists of eight arrays of >15,000 elements each, permitting comparison of two treatments with four-fold replication on each slide.

## Discussion

### Genomic Comparisons

Our identification of 26,669 *M. persicae *ESTs (Additional file 3) from 16 cDNA libraries extends previous sequencing efforts for this species [27] 25-fold, and contributes to the rapidly expanding resources that are available for aphid genomics. In addition to the described *M. persicae *data, the GenBank database contains 66,298 ESTs for *Acyrthosiphon pisum *(pea aphid; [36]), 8344 for *Aphis gossypii *(cotton aphid; [37]), 4263 for *Toxoptera citricida *(brown citrus aphid; [38]), 959 for *M. persicae *[27], and 458 for *Rhopalosiphon padi *(bird cherry-oat aphid; [39]). Sequencing of the *A. pisum *genome is ongoing [36], and a comprehensive database for all aphid genomics information has been established (; [40]). Functional analysis of aphid genes that are identified by sequencing or expression studies will be facilitated by the recent demonstration that it is possible to silence aphid gene expression by RNA interference [41,42].

The broad selection of source material for cDNA library construction (Table 3) permitted sequencing of ESTs representing genes expressed at different developmental stages and morphs, as well as genes expressed in response to viral infection, and alternate host plant utilization. In addition, the production of separate libraries from heads, digestive tracts, and salivary glands ensured that genes of special interest to the study of plant-aphid interactions are well-represented in our database. Our comparison of EST frequencies between non-normalized libraries enabled *in silico *prediction of differential gene expression (Table 5). Clustering of the ESTs from our first few libraries (Figure 1) indicated a high degree of redundancy. We responded to this by normalizing all subsequent libraries, which significantly increased our rate of new gene discovery but eliminated our ability to make inferences about differential expression between libraries. Therefore, it was advantageous to our project to make both types of library, preserving in some cases the natural transcript ratios present in the source tissues, and in others bringing the representation of housekeeping genes more in line with that of rarely expressed transcripts.

### Virus-derived Genes

Because aphids transmit plant viruses, and are themselves infected by entomopathogenic viruses, we searched our database for sequences with homology to known viral genes. No ESTs with homology to *Potato leafroll virus *(PLRV) were identified in our database, even in libraries made from aphids feeding on PLRV-infected plants. This absence of PLRV cDNA sequences is consistent with the fact that PLRV does not replicate within the aphids.

Five contigs are annotated as densovirus proteins, including one predicted to be specific to the aphid digestive tract (Table 5). All but one of the densovirus ESTs are from the USDA lineage, but this could well be an artifact relating to the fact that the gut cDNA library was made from this aphid strain. A densovirus has been reported to infect the anterior portion of the digestive tract of *M. persicae*, with infected aphids characterized by reduced size, delayed development, and decreased fecundity [32]. Densoviruses represent potential biological pest control agents, and similar viruses from the families *Baculoviridae *and *Tetraviridae *have been commercialized for this purpose.

When the stringency of the BlastX search was reduced to an E-value cutoff of 1E-4, one unigene (contig 3464, Additional file 2) has a Dasheen mosaic virus (DsMV) polyprotein as its best hit. DsMV is a non-persistent RNA virus known to be transmitted by *M. persicae *[43]. Over two-thirds of the 25 ESTs in the contig homologous to the DsMV polyprotein are derived from the salivary gland or head libraries, consistent with the fact that these non-circulative viruses are retained within the mouthparts of their aphid vectors. The relatively large number of DsMV-derived ESTs, which were found in seven different libraries from three lineages (F001, G006, and USDA), is unexpected in light of the fact that this virus should not replicate within the aphids, and none of the plants used for aphid rearing showed obvious signs of viral infection. Furthermore, the host range of DsMV is not known to overlap with the host plants used in these experiments.

### Functional significance of annotated unigenes

In cruciferous plants, myrosinase enzymes (β-thioglucosidases, EC 3.2.1.147) initiate the rapid breakdown of glucosinolates into insect-deterrent hydrolysis products during herbivory. However the aphids *Brevicoryne brassicae *(cabbage aphid) and *Lipaphis erysimi *(turnip aphid) have co-opted this defensive system by sequestering plant-derived glucosinolates and producing their own myrosinase as a defense against predators [44-47]. One EST from our database (accession number ES221351, from the G006 lineage) has significant homology to the *B. brassicae *myrosinase gene. Although attempts to measure myrosinase activity in *M. persicae *have been unsuccessful, it is notable that aliphatic rather than indole glucosinolates were used as enzymatic substrates in these experiments [48]. Aliphatic glucosinolates are recovered intact in the honeydew of *M. persicae *on *A. thaliana*, showing that these aphids are able to avoid or inactivate plant myrosinases. In contrast, *A. thaliana *indole glucosinolates are largely broken down within the aphids [49]. Although this glucosinolate breakdown may occur by a non-enzymatic mechanism, it is also possible that *M. persicae *possesses a myrosinase activity that is specific to indole rather than aliphatic glucosinolates.

The genetic mechanisms regulating the cyclically parthenogenetic life cycle characteristic of most aphids are largely unknown. Environmental cues, including shortening days, triggers development of sexual morphs in the autumn [50]. A gene from *A. pisum*, ApSD1, with similarity to a protein involved in amino acid transport in GABAergic neurons, is upregulated in pea aphids reared under short photoperiod conditions [51]. We identified one EST, which we had annotated as an amino acid transporter, as being significantly similar to ApSD1. This EST (accession number EC388175) was sequenced from the G006 male library, which is consistent with a role for this amino acid transporter in the development of winged sexual morphs.

*M. persicae *has evolved to tolerate plant allelochemicals and insecticides by diverse strategies, including amplification of E4 esterase genes [52], point mutations in insecticide targets [53], and increased activity of glutathione S-transferases in response to glucosinolates in artificial diets [33]. Out of 11 contigs with significant homology to *M. persicae *esterases, two contigs (720 and 3118) from our database are nearly identical to the *M. persicae *E4 esterase (GenBank Accession CAA52648), whereas nine others appear to represent different genes. These nine sequences may have evolved following amplification to acquire novel functions in the hydrolysis of plant secondary metabolites encountered during the expansion of the insect's host range, or in the breakdown of newly developed insecticides. Other potential detoxification genes represented in our database include 24 glutathione S-transferases and 53 cytochrome P450s (Additional file 2).

Among the 168 salivary gland contigs that are predicted to encode secreted proteins (Additional file 3), approximately 62% are of unknown function. However, others could have potential function in aphid virulence based on their homology to known proteins. For instance, contig 1300 encodes a protein that belongs to an insect-specific family that includes the yellow proteins of *D. melanogaster*, that are involved in cuticular development and behavior [54], and the major royal jelly proteins of *Apis mellifera *(honeybee). *A. mellifera *proteins from this family are high in essential amino acids and comprise up to 90% of the total protein content of the jelly that is fed to developing larvae [55]. Although major royal jelly proteins are thought to be produced in the cephalic glands of nurse bees [56], another member of this protein family (MRJP 8) was recently identified as a component of the honeybee venom [57]. In *M. persicae*, the homologous protein is less abundant, and ESTs were only found in the salivary gland and normalized head libraries (MpSG and MpHnorm in Table 3). Nevertheless, it is tempting to speculate that the protein has a virulence function in aphids. Two other genes expressed in salivary glands, represented by contigs 2422 and 3025, are predicted to encode secreted proteins that play a role in proteolysis, and therefore could have interesting functions in the interaction between *M. persicae *and its host plants. Contig 2422, which has highest homology to a sequence of unknown function from *D. melanogaster *(GenBank accession NP_611740), encodes a protease-associated domain. Contig 3025 encodes a protein with homology to *Der1*, a gene involved in the degradation of misfolded proteins in yeast [58].

### DNA Sequence Polymorphisms

Comparison of ESTs from the three *M. persicae *lineages identified a large number of potential sequence polymorphisms which were subjected to stringent post-processing to reduce sequencing artifacts. The remaining 167 SNPs, represented by multiple ESTs in more than one aphid lineage (Additional file 6), are a good data source for the identification of *M. persicae *genetic markers. Furthermore, as suggested by the cathepsin B-N sequence data (Figure 3), these polymorphisms may provide clues about functional divergence of proteins in different *M. persicae *lineages.

However, when we re-sequenced 11 of these SNPs from genomic DNA templates, only about half were confirmed (Table 6), suggesting that many potential sequence differences in our EST collection are the result of errors created during reverse transcription, PCR amplification, or sequencing. This highlights the importance of developing effective criteria to select a list of high-confidence SNPs from the large number of polymorphisms predicted by programs such as POLYBAYES, and of validating predicted polymorphisms by re-sequencing of genomic DNA.

### Microarray Development

Given the greater reproducibility of gene expression data collected with oligonucleotide microarrays, as opposed to spotted cDNA microarrays, we decided to develop oligonucleotide microarrays for future studies on *M. persicae *gene expression [59]. The highest quality microarrays currently available are those fabricated by *in situ *oligonucleotide synthesis, a technology pioneered by Agilent. When using such arrays, the number of required technical replicates is reduced because of the high degree of reproducibility between spots, allowing the user to concentrate resources on analyzing biological replicates. In addition, the high cost of purchasing synthesized oligonucleotides makes traditional custom printing of high density arrays at core facilities feasible only if many arrays will be made. There are no up-front costs to design microarrays on Agilent's eArray platform, and the minimum number of slides to order is one.

Transcriptional profiling with microarrays is a powerful technique for identifying genes involved in the response of an organism to its environment. We anticipate that *M. persicae *microarrays can be used to answer a variety of fundamental questions about aphid biology and plant-aphid interactions. Genes critical to the status of this insect as an agricultural pest can be identified by studying expression changes induced by different crop plants and in response to virus infection. Research on aphid genes specifically expressed in salivary glands may identify proteins that prevent clogging of sieve elements or otherwise contribute to the phloem-specific feeding style of aphids. Conversely, these salivary proteins likely also provide phloem-specific cues that allow plants to recognize aphid feeding and mount a defense response. Microarray experiments will allow association of gene expression changes with polyphenism, the development for morphologically different individuals (*e.g*. winged and unwinged) that are otherwise genetically identical. Analysis of gene expression in aphids feeding on artificial diets or plants with altered amino acid content can identify genes that are critical for the interaction with endosymbiotic *B. aphidicola *bacteria, which synthesize essential amino acids and allow aphids to survive on the otherwise nutritionally imbalanced phloem sap.

The broad host range and differences in host plant preferences among individual lineages of *M. persicae *are some of the more interesting aspects of the biology of this insect. Gene expression differences that underlie within-species variation can be identified by microarray analysis. By sequencing cDNA libraries made from aphids that were raised on both Solanaceae and Cruciferae, we have increased the probability that future microarray experiments performed by ourselves and others will include aphid genes that are expressed only under these particular growth conditions. Evidence for such regulated gene expression comes from our non-normalized libraries, which included two genes that were overrepresented among ESTs from *N. benthamiana *in comparison to *A. thaliana *(Table 5). DNA microarray experiments will almost certainly identify additional genes with host plant specific expression patterns. Further research on the function of such differentially expressed genes will illuminate adaptations that have allowed some *M. persicae *lineages to expand their host range to include tobacco. Other *M. persicae *lineages, which show differences in their ability to reproduce on *A. thaliana *(J. Kim and G. Jander, unpublished results), can be studied to identify aphid adaptations for feeding on Cruciferae. In addition, microarray experiments with *M. persicae *feeding on *A. thaliana *will provide the unique opportunity to simultaneously study gene expression changes on both sides of a plant-insect interaction.

Given the broad range of questions that can be addressed by microarray analysis of *M. persicae *gene expression, the Agilent microarray that we have developed will be of broad interest to aphid researchers. Although the technology necessary for hybridizing and scanning synthesized Agilent arrays is somewhat different from that used for experiments with spotted oligonucleotide arrays, it is available at many universities. The microarrays described here will be made available at cost to other researchers and can be obtained by contacting the corresponding author (G.J.).

## Conclusion

By sequencing and analyzing 26,669 *M. persicae *ESTs, we have generated new genomic resources for this aphid species. Expressed aphid genes, in particular many that show no significant similarity to genes from other organisms, have been identified. Molecular markers that were found by comparing three aphid lineages will be useful not only for genotyping natural isolates, but also future genetic studies with *M. persicae*. The DNA microarray that has been developed will permit further investigation of agriculturally and ecologically relevant transcriptional regulation in *M. persicae*.

To date, the lack of genomic resources for *M. persicae *has stood in stark contrast to the threat posed by this aphid to agricultural systems worldwide. By studying aphid gene expression responses to virus infection, different host plants, and other stresses, it will be possible to obtain a better understanding of this important biological interaction. In addition, our increasing understanding of plant molecular responses to phloem-feeding insects will be complemented by elucidation of the adaptations that allow these insects to establish compatible interactions with their host plants. Further research on *M. persicae *gene expression responses will aid in efforts to breed crops with increased aphid resistance and will advance ongoing research into aphid ecology, evolution, and physiology.

## Methods

### Aphid Collection, Rearing, and Characterization

*M. persicae *lineages were collected from five sites in the United States and one in Scotland, as described in Table 1 and Additional file 1. Aphid lineages were started from a single greenhouse- or field-collected insect. Colonies were reared on *B. oleracea *var. 'Wisconsin Golden Acres' in growth chambers (16:8 h light:dark cycle, 150 μmols m^-2^s^-1^, at 24°C ± 1 day, 19°C ± 1 night, 50% relative humidity). For library construction, aphids from the USDA lineage were reared under the same conditions on *A. thaliana *(land race Columbia-0), *N. benthamiana*, and *B. oleracea *var. 'Wisconsin Golden Acres'. Asexual females of lineages F001 and G006 were reared on PLRV-infected or PLRV-free *P. floridana *(15:9 h light:dark cycle at 24°C). For induction of sexual morphs, aphids were reared on *B. oleracea *var 'Wisconsin Golden Acre' in growth chambers under short day conditions (13:11 h light:dark cycle, 115 μmol/m^2^/s light intensity, 18°C ± 2) in Percival (Perry, IA, USA) Model I36LLVLC8 growth chambers. The 2001–12 lineage was maintained on *B. napus *var. 'Mascot' (16:8 h light:dark cycle at 18°C ± 2).

*M. persicae *lineages were genotyped in a single multiplex PCR reaction containing dye-labelled primers (Additional file 7) to amplify seven microsatellite loci: M86 and M40 [25] and S17b, myz2, myz3, myz9 and myz25 [26]. One primer for each locus was fluorescently labeled with the following dyes; NED™: S17b-forward and myz25-forward, 6-FAM™: M86-forward, VIC™: myz3-forward, myz9-forward, M40-forward and PET™: myz2-reverse (Applied Biosystems, Foster City, California, USA). PCR reactions were carried out with 10 μl reaction volumes containing 0.5 units of *Taq *DNA polymerase (Eppendorf, Hamburg, Germany), 50 mM KCl, 10 mM Tris-HCL pH 8.3, 2.0 mM Mg^2+^, 200 μM of each dNTP, 125 pM of primers myz2f, myz2r, myz9f, myz9r, M40f, M40r, M86f, M86r, myz3f and myz3r, 62.5 pM of primers S17bf, S17br, myz25f and myz25r and approximately 5 ng of template DNA. PCR reactions were run using 5-dye chemistry on an ABI 3730 DNA Analyzer (Applied Biosystems, Foster City, CA, USA) by the Genomic Analysis and Technology Core facility at the University of Arizona. Fragment analysis was completed using STRand software .

For life cycle characterization, about ten months after their field collection, three parthenogenetic lines of each aphid lineage were established on cabbage seedlings in small plexiglass cages. Aphids were raised on these plants as separate lines for three generations at 20°C long day (16:8 h light:dark cycle) to remove maternal and grand-maternal effects. Three third-generation (G3) adults were transferred to a new plant and placed at short day conditions (10:14 h light:dark cycle) at 15°C. After three days the adults were removed and the juveniles were returned to short day conditions. When the G4 aphids were third or fourth instars, three aphids per line were transferred to a new plant and returned to the cabinet under inducing conditions to give birth to the first batch of G5 individuals (G5-1). One week later the three G4 adults were transferred to a new plant to give birth to the G5 batch 2 individuals (G5-2). This processes was repeated a third time to generate the G5 batch 3 progeny. The adult morphs of the three batches of G5 progeny from each line were scored. Lineages that produced males and pre-sexual females (gynoparae) and in at least one of the three replicates and no asexual females (vivipara) in any of the three replicate lines were classified as cyclical parthenogens (holocyclics). Lineages that produced all three morphs males, gynoparae and vivipara were classified as intermediates. Lineages that produced males in at least one replicate and vivipara but no gynoparae were classified as androcyclics and finally lineages that failed to produce any sexual morphs were classified as obligate parthenogens (anholocyclics). Gynoparae are winged females and were distinguished from alate viviparous aphids by the exclusively production of sexual female progeny (ovipara).

Holocyclic *M. persicae *lineages were tested for their ability to transmit PLRV. A large quantity (>200) of aphids reared on healthy *P. floridana *plants was placed in a dish containing *P. floridana *leaves infected with PLRV. Aphids were allowed to feed for a 48 hour acquisition period before being transferred to healthy young *P. floridana *plants. After a 5-day transmission period, plants were treated with insecticide (Dibrom 8E; Valent, Walnut Creek, CA, USA) and transferred to the greenhouse. Symptoms of PLRV were observed within three to five weeks.

### Tissue Collection

Three separate aphid tissues (digestive tracts, heads, and salivary glands) were isolated from the USDA red lineage. Digestive tracts and heads were dissected from alate adult asexual females that had been anesthetized by dipping each individual in 70% ethanol. Dissections were performed using a pin embedded in a wooden handle that was inserted dorsally between the head and thorax while holding aphids by their wings with forceps. Aphid heads with the intact digestive tract attached were removed by applying light pressure anteriorly with the pin and posteriorly with the forceps. Following dissection, head and gut tissue were separated and stored individually in RNAlater (Ambion, Austin, TX, USA) at -20°C for up to one month. Salivary gland tissue was dissected from ~400 aphids of different life stages (predominantly fourth instar alates and alate adults) reared on cabbage (*B. oleraceae*). Aphid heads were detached from their bodies as described above, and then the salivary glands were exposed by removal of the antennae, stylet, and head capsule. Following this, both sets of principal and accessory glands were carefully removed from the remaining tissues and stored in RNAlater (Ambion, Austin, TX, USA) solution.

Adult males were obtained after approximately six weeks and adult sexual females after seven weeks following transfer to short day conditions (13:11 h light:dark cycle at 18°C ± 2). Altogether, 92 sexual females and 128 males of lineage F001, and 81 sexual females and 134 males of lineage G006 were flash frozen and stored at -80°C for library construction.

### cDNA Library Construction

Total RNA was isolated using RNeasy kits (Qiagen, Valencia, CA, USA) or Tripure reagant (Roche, Indianapolis, IN, USA) and purified mRNA using Oligotex resin (Qiagen, Valencia, CA, USA) or Dynabeads mRNA Purification Kit (Invitrogen, Carlsbad, CA, USA). All cDNA libraries were made from mRNA with the exception of the salivary gland library, (MpSG), which was made from total RNA. Genomic DNA was isolated from flash frozen aphids following the "salting-out" protocol[60].

#### Non-normalized libraries

Four libraries were made following the LD PCR protocol from the Creator SMART cDNA Library Construction Kit (Clontech, Mountain View, CA, USA). cDNA generated by reverse transcription was amplified, digested with *Sfi *1A and *Sfi *1B, and size fractionated. Double-stranded cDNA was directionally cloned into the pDNR-LIB plasmid vector, and transformed into DH10B competent cells (Invitrogen, Carlsbad, CA, USA). The cDNA for a fifth non-normalized library was generated from mRNA and cloned using a Superscript Plasmid system with Gateway technology (Invitrogen, Carlsbad, CA, USA). For this library, the size fractionated cDNA was cloned into the pSPORT1 vector cut with *Not*I and *Sal*I and the recombinant plasmids were used to transform ElectroMAX DH10B cells (Invitrogen, Carlsbad, CA, USA).

#### Normalized libraries

Eleven normalized cDNA libraries were constructed using the TRIMMER direct cDNA Normalization kit (Evrogen, Moscow, Russia) in conjunction with the Creator SMART kit. We generated cDNA by reverse transcription of total RNA (salivary gland library) and mRNA (all other libraries). Double-stranded cDNA for normalization was generated using 15–21 PCR cycles. The double-stranded cDNA was denatured and allowed to re-hybridize under stringent conditions; subsequently the reaction mixture was treated with a duplex specific nuclease [61]. The duplexes corresponded disproportionately to abundant cDNAs, leaving a population of single-stranded cDNA molecules in which the representation of rare transcripts was increased. The remaining single stranded cDNA molecules were subsequently amplified, and library construction proceeded as for non-normalized libraries.

### EST Sequencing

Sequencing reactions were performed either on purified plasmids or on PCR-amplified products.

#### PCR-amplified products

Library aliquots were spread onto selective media and grown overnight at 37°C. Colonies were picked manually into 384 well plates (Genetix, New Milton, Hampshire, UK) containing selective media and grown overnight at 37°C. One μL of liquid culture was used as a template for colony PCR (primer sequences in Additional file 7). Colony PCR products were analyzed by gel electrophoresis to confirm the presence of an insert. PCR products were purified using MinElute 96 UF plates (Qiagen, Valencia, CA, USA) or AMpure (Agencourt Biosciences, Beverly, MA, USA). Sequencing reactions were carried out using ABI PRISM BigDye technology, and sequences were analyzed on the ABI 3730XL automated multicapillary sequencer (Applied Biosystems, Foster City, CA, USA).

#### Purified plasmids

Library aliquots were spread onto Q-tray vented bioassay plates (Genetix, New Milton, Hampshire, UK) containing selective media and grown for 18 hours at 37°C. Colonies were picked by the Qbot robotic colony manipulator (Genetix, New Milton, Hampshire, UK) into 384-well plates containing selective media and grown for 12 hours at 37°C. Plasmid DNA was purified using SprintPrep 384 HC kits (Agencourt Biosciences, Beverly, MA, USA) and subject to dye-terminator fluorescent DNA sequencing. The sequencing products were purified using CleanSEQ (Agencourt Biosciences, Beverly, MA, USA), and the sequences were analyzed on the ABI 3730XL (Applied Biosystems, Foster City, CA, USA) automated multicapillary sequencer.

### Sequence Processing and Annotation

We used Phred [62,63] to make base calls from sequence traces. Vector and adaptor sequences were identified from each EST using Crossmatch [63], and trimmed along with poly-A tails and low quality sequence (*i.e*. 10 or more bases out of 25 with a quality score below 20). ESTs containing less than 100 bases of quality sequence were discarded. All ESTs were compared to the GenBank nr database using BlastN [64]. Those ESTs for which the best Blast targets were *B. aphidicola *sequences with E-value less than 1E-20 were considered to be endosymbiont contamination and were filtered out. ESTs passing quality tests were clustered using the TribeMCL software (1E-50 and 95% identity for Blast alignment; inflation value 5; [65], and consensus contig sequences were generated using Cap3 [66].

For functional annotation we used the Gene Ontology annotation from the NCBI Gene database (Maglott et al., 2005). For classification purposes, we converted all the GO terms in the Gene database to GOslim [29]. BlastX was used to match the contig sequences to the NCBI refseq protein sequences. GOslim annotations of the Refseq hits with BlastX e-value less than 1E-5 were transferred to the query contigs.

### SNP Identification and Confirmation

The PolyBayes program [34] was used to identify SNPs. Consensus contig sequences were used as anchor sequences for each alignment, and the cutoff for the SNP probability score was 0.84. Seven PCR primer pairs were used to amplify the 11 predicted SNPs (contigs 129, 254, 1080, 3202, 3285, 3347, 3713: Additional file 7) from the F001, G006 and USDA lineages. Primers for elongation factor 1α (EF-1α) were used in control PCR reactions. PCR products were purified using Ampure (Agencourt Biosciences, Beverly, MA, USA). Sequencing reactions were carried out using ABI PRISM BigDye technology (Applied Biosystems, Foster City, CA, USA), and sequences were analyzed on an ABI 3730XL automated multicapillary sequencer. Sequences were aligned using ClustalW [67] to confirm the presence of the putative polymorphism.

### *In silico *Prediction of Tissue-Specific Gene Expression

The method proposed by [31] was used for comparing gene expression profiles from four of the non-normalized cDNA libraries. For each contig, the number of ESTs from each library was counted and the R statistic was calculated. Potential salivary gland specific ESTs were also identified from the normalized salivary gland library. Contigs considered likely to be expressed in salivary glands contained at least 2 ESTs from the MpSG library, and at least 50% of the ESTs from those contigs were derived from the MpSG library. For each contig, the probability of meeting the selection criteria was calculated using the binomial distribution, the percentage of all ESTs that were in salivary gland libraries (12.1%), and the number of ESTs that contributed to the given contig. Predicted open reading frames from potential salivary gland contigs were translated *in silico *using the ExPASy translator tool [68]. The predicted protein sequence was run through SignalP 3.0 [69], using both the neural network and Hidden Markov model to identify a possible signal peptide in the predicted proteins.

### Microarray Design

A 15,000-element expression array containing 60-mers representing the identified *M. persicae *unigene set was designed using eArray (Agilent, Santa Clara, CA, USA). Arrays are being produced at Agilent by *in situ *oligonucleotide synthesis.

## List of Abbreviations

Bt: *Bacillus thuringiensis*

EST: Expressed sequence tag

PLRV: *Potato leafroll virus*

SNP: Single nucleotide polymorphism

## Authors' contributions

ACCW, BF, and GJ collected aphid strains. ACCW, BF, GJ, JSR, MdV, and SG designed experiments. ACCW characterized aphid life cycles and collected sexual morphs. DS and SG conducted virus transmission experiments. AW, MdV, GM, and JSR constructed and sequenced cDNA libraries. ACCW, AW, BF, CT, GJ, GM, JSR, MdV, and QS conducted bioinformatic data analysis. ACCW, GJ, JSR, and MdV wrote the manuscript. All authors read and approved the final manuscript.

## Supplementary Material

Additional file 1 Collection and life cycle characterization of aphid lineages. This table provides information on aphids collected for this study, including host plant and site of collection, and results of life cycle characterization. Click here for file

Additional file 2 BlastX comparisons of *M. persicae *contigs to GenBank DNA sequences. This table provides a working annotation of unigenes described in this study, based on BlastX comparisons to GenBank DNA sequences. Click here for file

Additional file 3 Full listing of *M. persicae *ESTs, along with the source cDNA libraries. This table lists accession numbers of all ESTs sequenced in this study, in association with the contig to which they belong and the library from which they are derived. Click here for file

Additional file 4 Secreted proteins in salivary glands, as predicted by SignalP 3.0. This table provides annotation and signal peptide prediction information for unigenes overrepresented in the salivary gland library. Click here for file

Additional file 5 Potential SNPs identified based on sequence differences among *M. persicae *lineages. This table lists SNPs identified among the sequences generated in this study by PolyBayes with a probability cutoff of 0.84. Click here for file

Additional file 6 High-confidence SNPs, represented by at least two reads in at least two *M. persicae *lineages. This table provides a list of filtered SNPs likely to represent polymorphisms between *M. persicae *lineages. Click here for file

Additional file 7 PCR primers used in this study. This table lists the sequences for all PCR primers used in this study. Click here for file

## References

[B1] Robertson MJ, Zehnder GW, Hammig MD (2005). Adaptation of integrated pest management practices by South Carolina cotton growers. Journal of Extension.

[B2] Girousse C, Moulia B, Silk W, Bonnemain JL (2005). Aphid infestation causes different changes in carbon and nitrogen allocation in alfalfa stems as well as different inhibitions of longitudinal and radial expansion. Plant Physiology.

[B3] Pegadaraju V, Knepper C, Reese J, Shah J (2005). Premature leaf senescence modulated by the Arabidopsis PHYTOALEXIN DEFICIENT4 gene is associated with defense against the phloem-feeding green peach aphid. Plant Physiol.

[B4] Gray SM, Gildow F (2003). Luteovirus-Aphid Interactions. Annu Rev Phytopathol.

[B5] Kennedy JS, Day MF, Eastop VF (1962). A Conspectus of Aphids as Vectors of Plant Viruses.

[B6] Blackman RL, Eastop VF (2000). Aphids on the World's Crops.

[B7] Mowry TM (2005). Insecticidal reduction of Potato leafroll virus transmission by Myzus persicae.. Annals of Applied Biology.

[B8] Terradot L, Simon JC, Leterme N, Bourdin D, Wilson ACC, Gauthier JP, Robert Y (1999). Molecular characterization of clones of the Myzus persicae complex (Hemiptera: Aphididae) differing in their ability to transmit potato leafroll luteovirus. Bull Ent Res.

[B9] Vorburger C, Lancaster M, Sunnucks P (2003). Environmentally related patterns of reproductive mode is the aphid Myzus persicae and the predominance of two superclones in Victoria, Australia.. Molecular Ecology.

[B10] Zitoudi K, Margaritopoulos JT, Mamuris Z, Tsitsipis JA (2001). Genetic variation in Myzus persicae populations associated with host-plant and life cycle category.. Ent Exp Appl.

[B11] Blackman RL (1987). Morphological discrimination of a tobacco-feeding form from Myzus persicae (Sulzer) (Hemiptera: Aphididae), and a key to New World Myzus (Nectarosiphon) species. Bull Ent Res.

[B12] Guthrie FE, Campbell WV, Baron RL (1962). Feeding sites of the green peach aphid with respect to its adaptation to tobacco. Ann Ent Soc Am.

[B13] McPherson RM (1989). Seasonal abundance of red and green morphs of the tobacco aphid (Homoptera: Aphididae) on flue-cured tobacco in Georgia. J Ent Sci.

[B14] Lampert EP, Dennis CA (1987). Life history of two color morphs of the green peach aphid (Homoptera: Aphididae) on flue-cured tobacco. Tobacco Sci.

[B15] Eastop VF, Blackman RL (2005). Some new synonyms in Aphididae (Hemiptera: Sternorrhyncha).. Zootaxa.

[B16] de Vos M, van Oosten VR, van Poecke RM, van Pelt JA, Pozo MJ, Mueller MJ, Buchala AJ, Metraux JP, van Loon LC, Dicke M, Pieterse CM (2005). Signal signature and transcriptome changes of Arabidopsis during pathogen and insect attack. Mol Plant Microbe Interact.

[B17] Moran PJ, Cheng Y, Cassell JL, Thompson GA (2002). Gene expression profiling of Arabidopsis thaliana in compatible plant-aphid interactions. Arch Insect Biochem Physiol.

[B18] Couldridge C, Newbury HJ, Ford-Lloyd B, Bale J, Pritchard J (2007). Exploring plant responses to aphid feeding using a full Arabidopsis microarray reveals a small number of genes with significantly altered expression. Bull Ent Res.

[B19] Pegadaraju V (2005). Molecular insights into Arabidopsis response to Myzus persicae Sulzer (green peach aphid). Biology.

[B20] Kusnierczyk A, Winge P, Midelfart H, Armbruster WS, Rossiter JT, Bones AM (2007). Transcriptional responses of Arabidopsis thaliana ecotypes with different glucosinolate profiles after attack by polyphagous Myzus persicae and oligophagous Brevicoryne brassicae.. Journal of Experimental Botany.

[B21] Ruiz MT, Voinnet O, Baulcombe DC (1998). Initiation and maintenance of virus-induced gene silencing. Plant Cell.

[B22] Bhattarai KK, Li Q, Liu Y, Dinesh-Kumar SP, Kaloshian I (2007). The MI-1-mediated pest resistance requires Hsp90 and Sgt1. Plant Physiol.

[B23] Lee L, Palukaitis P, Gray SM (2002). Host-dependent requirement for the Potato leafroll virus 17-kda protein in virus movement.. Mol Plant Microbe Interact.

[B24] Finston TL, Hebert DN, Foottit RB (1995). Genome size variation in aphids. Insect Biochem Molec Biol.

[B25] Sloane MA, Sunnucks P, Wilson AC, Hales DF (2001). Microsatellite isolation, linkage group identification and determination of recombination frequency in the peach-potato aphid, Myzus persicae (Sulzer) (Hemiptera: Aphididae). Genet Res.

[B26] Wilson ACC, Massonnet B, Simon JC, Prunier-Leterme N, Dolatti L, Llewellyn KS, Figueroa CC, Ramirez CC, Blackman RL, Estoup A, Sunnucks P (2004). Cross-species amplification of microsatellite loci in aphids: assessment and application. Molecular Ecology Notes.

[B27] Figueroa CC, Prunier-Leterme N, Rispe C, Sepulveda F, Fuentes-Contreras E, Sabater-Munoz B, Simon JC, Tagu D (2007). Annotated expressed sequence tags and xenobiotic detoxification in the aphid Myzus persicae (Sulzer). Insect Science.

[B28] Fenton B, Malloch G, Woodford JAT, Foster SP, Anstead J, Denholm I, King L, Pickup J (2005). The attack of the clones: tracking the movement of insecticide-resistant peach–potato aphids Myzus persicae (Hemiptera: Aphididae). Bull Ent Res.

[B29] Harris MA, Clark J, Ireland A, Lomax J, Ashburner M, Foulger R, Eilbeck K, Lewis S, Marshall B, Mungall C, Richter J, Rubin GM, Blake JA, Bult C, Dolan M, Drabkin H, Eppig JT, Hill DP, Ni L, Ringwald M, Balakrishnan R, Cherry JM, Christie KR, Costanzo MC, Dwight SS, Engel S, Fisk DG, Hirschman JE, Hong EL, Nash RS, Sethuraman A, Theesfeld CL, Botstein D, Dolinski K, Feierbach B, Berardini T, Mundodi S, Rhee SY, Apweiler R, Barrell D, Camon E, Dimmer E, Lee V, Chisholm R, Gaudet P, Kibbe W, Kishore R, Schwarz EM, Sternberg P, Gwinn M, Hannick L, Wortman J, Berriman M, Wood V, de la Cruz N, Tonellato P, Jaiswal P, Seigfried T, White R (2004). The Gene Ontology (GO) database and informatics resource. Nucleic Acids Res.

[B30] Pruitt KD, Tatusova T, Maglott DR (2007). NCBI reference sequences (RefSeq): a curated non-redundant sequence database of genomes, transcripts and proteins. Nucleic Acids Res.

[B31] Stekel DJ, Git Y, Falciani F (2000). The comparison of gene expression from multiple cDNA libraries. Genome Res.

[B32] van Munster M, Dullemans AM, Verbeek M, van den Heuvel JF, Reinbold C, Brault V, Cleriver A, van der Wilk F (2003). Characterization of a new densovirus infecting the green peach aphid Myzus persicae. Journal of Invertebrate Pathology.

[B33] Francis F, Vanhaelen N, Haubruge E (2005). Glutathione S-transferases in the adaptation to plant secondary metabolites in the Myzus persicae aphid. Arch Insect Biochem Physiol.

[B34] Marth GT, Korf I, Yandell MD, Yeh RT, Gu Z, Zakeri H, Stitziel NO, Hillier L, Kwok PY, Gish WR (1999). A general approach to single-nucleotide polymorphism discovery. Nat Genet.

[B35] Bennett-Lovsey RM, Herbert AD, Sternberg MJE, Kelley LA (2007). Exploring the extremes of sequence/structure space with ensemble fold recognition in the program Phyre. Proteins: Structure, Function and Bioinformatics.

[B36] Sabater-Munoz B, Legeai F, Rispe C, Bonhomme J, Dearden P, Dossat C, Duclert A, Gauthier JP, Ducray DG, Hunter W, Dang P, Kambhampati S, Martinez-Torres D, Cortes T, Moya A, Nakabachi A, Philippe C, Prunier-Leterme N, Rahbe Y, Simon JC, Stern DL, Wincker P, Tagu D (2006). Large-scale gene discovery in the pea aphid Acyrthosiphon pisum (Hemiptera). Genome Biol.

[B37] Lee L, Hunter WB, Hunnicutt LE, Dang PM (2005). An expressed sequence tag (EST) cDNA library of Aphis gossypii alates: ; Austin, TX..

[B38] Hunter WB, Dang PM, Bausher MG, Chaparro JX, McKendree W, Shatters RG, McKenzie CL, Sinisterra XH (2003). Aphid biology: expressed genes from alate Toxoptera citricida, the brown citrus aphid. J Insect Sci.

[B39] Tagu D, Prunier-Leterme N, Legeai F, Gauthier JP, Duclert A, Sabater-Muñoz B, Bonhomme J, Simon JC (2004). Annotated expressed sequence tags for studies of the regulation of reproductive modes in aphids. Insect Biochem Mol Biol.

[B40] Gauthier JP, Legeai F, Zasadzinski A, Rispe C, Tagu D (2007). AphidBase: a database for aphid genomic resources. Bioinformatics.

[B41] Mutti NS, Park Y, Reese JC, Reeck GR (2005). RNAi knockdown of a salivary transcript leading to lethality in the pea aphid, Acyrthosiphon pisum. J Insect Sci.

[B42] Jaubert S, Le Trionnaire G, Bonhomme J, Christophides GK, Rispe C, Tagu D (2007). Gene knockdown by RNAi in the pea aphid Acyrthosiphon pisum.. BMC Biotechnology.

[B43] Ram R, Joshi A, Verma N, Kulshrestha S, Raikhy G, Hallan V, Zaidi AA (2003). First report of Dasheen mosaic virus infecting four ornamental aroids in India. Plant Path.

[B44] Pontoppidan B, Ekbom B, Eriksson S, Meijer J (2001). Purification and characterization of myrosinase from the cabbage aphid (Brevicoryne brassicae), a Brassica herbivore. Eur J Biochem.

[B45] Jones AM, Bridges M, Bones AM, Cole R, Rossiter JT (2001). Purification and characterisation of a non-plant myrosinase from the cabbage aphid Brevicoryne brassicae (L.). Insect Biochem Mol Biol.

[B46] Francis F, Lognay G, Wathelet JP, Haubruge E (2002). Characterisation of aphid myrosinase and degradation studies of glucosinolates. Arch Insect Biochem Physiol.

[B47] Francis F, Lognay G, Wathelet JP, Haubruge E (2001). Effects of allelochemical from first (Brassicaceae) and second (Myzus persicae and Brevicornye brassicae) trophic levels on Adalia bipunctata. Journal of Chemical Ecology.

[B48] MacGibbon DB, Allison RM (1968). Glucosinolate system in the aphid Brevicoryne brassicae. New Zealand J Sci.

[B49] Kim JH, Jander G (2007). Myzus persicae (green peach aphid) feeding on Arabidopsis induces the formation of a deterrent indole glucosinolate. Plant Journal.

[B50] Tagu D, Sabater-Muñoz B, Simon JC (2005). Deciphering reproductive polyphenism in aphids. Inv Reprod Dev.

[B51] Ramos S, Moya A, Martinez-Torres D (2003). Identification of a gene overexpressed in aphids reared under short photoperiod. Ins Bioch Mol Bio.

[B52] Field LM, Foster SP (2002). Amplified esterase genes and their relationship with other insecticide resistance mechanisms in English field populations of the aphid, Myzus persicae (Sulzer). Pest Manag Sci.

[B53] Martinez-Torres D, Foster SP, Field LM, Devonshire AL, Williamson MS (1999). A sodium channel point mutation is associated with resistance to DDT and pyrethroid insecticides in the peach-potato aphid, Myzus persicae (Sulzer) (Hemiptera: Aphididae). Insect Mol Bio.

[B54] Maleszka R, Kucharski R (2000). Analysis of Drosophila yellow-B cDNA reveals a new family of proteins related to the royal jelly proteins in the honeybee and to an orphan protein in an unusual bacterium Deinococcus radiodurans. Biochem Biophys Res Commun.

[B55] Schmitzova J, Klaudiny J, Albert S, Schroder W, Schreckengost W, Hanes J, Judova J, Simuth J (1998). A family of major royal jelly proteins of the honeybee Apis mellifera L. Cell Mol Life Sci.

[B56] Drapeau MD, Albert S, Kucharski R, Prusko C, Maleszka R (2006). Evolution of the yellow/major royal jelly protein family and the emergence of social behavior in honey bees. Genome Res.

[B57] Peiren N, Vanrobaeys F, de Graaf DC, Devreese B, Van Beeumen J, Jacobs FJ (2005). The protein composition of honeybee venom reconsidered by a proteomic approach. Biochim Biophys Acta.

[B58] Knop M, Finger A, Braun T, Hellmuth K, Wolf DH (1996). Der1, a novel protein specifically required for endoplasmic reticulum degradation in yeast. Embo J.

[B59] Brennan C, Zhang Y, Leo C, Feng B, Cauwels C, Aguirre AJ, Kim M, Protopopov A, Chin L (2004). High-resolution global profiling of genomic alterations with long oligonucleotide microarray. Cancer Res.

[B60] Sunnucks P, Hales DF (1996). Numerous transposed sequences of mitochondrial cytochrome oxidase I-II in aphids of the genus Sitobion (Hemiptera: Aphididae). Molecular Biology and Evolution.

[B61] Zhulidov PA, Bogdanova EA, Shcheglov AS, Vagner LL, Khaspekov GL, Kozhemyako VB, Matz MV, Meleshkevitch E, Moroz LL, Lukyanov SA, Shagin DA (2004). Simple cDNA normalization using kamchatka crab duplex-specific nuclease. Nucleic Acids Res.

[B62] Ewing B, Green P (1998). Base-calling of automated sequencer traces using phred. II. Error probabilities. Genome Res.

[B63] Ewing B, Hillier L, Wendl MC, Green P (1998). Base-calling of automated sequencer traces using phred. I. Accuracy assessment. Genome Res.

[B64] Altschul SF, Gish W, Miller W, Myers EW, Lipman DJ (1990). Basic local alignment search tool. J Mol Biol.

[B65] Enright AJ, Van Dongen S, Ouzounis CA (2002). An efficient algorithm for large-scale detection of protein families. Nucleic Acids Res.

[B66] Huang X, Madan A (1999). CAP3: A DNA sequence assembly program. Genome Res.

[B67] Thompson JD, Higgins DG, Gibson DJ (1995). CLUSTALW: improving the sensitivity of progressive multiple sequence alignment through sequence weighting, position-specific gap penatilies and weight matrix choice. Nuc Acids Res.

[B68] Gasteiger E, Gattiker A, Hoogland C, Ivanyi I, Appel RD, Bairoch A (2003). ExPASy: The proteomics server for in-depth protein knowledge and analysis. Nucleic Acids Res.

[B69] Bendtsen JD, Nielsen H, von Heijne G, Brunak S (2004). Improved prediction of signal peptides: SignalP 3.0. J Mol Biol.

